# A New Multidisciplinary Model of Glomerulonephritis Care in Ontario: A Descriptive Program Report

**DOI:** 10.1177/20543581251394082

**Published:** 2025-12-01

**Authors:** Arrti A. Bhasin, Stephanie N. Dixon, Lavanya Bathini, Nivethika Jeyakumar, Lima F. Rodrigues, Yuguang Kang, Peter G. Blake, Amit X. Garg, Michelle A. Hladunewich

**Affiliations:** 1 Ontario Health (Ontario Renal Network), Toronto, Canada; 2 London Health Sciences Centre Research Institute, ON, Canada; 3 ICES, London, ON, Canada; 4 Department of Epidemiology and Biostatistics, Schulich School of Medicine & Dentistry, Western University, London, ON, Canada; 5 Department of Medicine, University of Alberta, Edmonton, Canada; 6 University of Alberta Hospital, Edmonton, Canada; 7 Division of Nephrology, Department of Medicine, Schulich School of Medicine and Dentistry, Western University, London, ON, Canada; 8 Division of Nephrology, Department of Medicine, Sunnybrook Health Sciences Centre, Toronto, ON, Canada; 9 Temerty Faculty of Medicine, University of Toronto, ON, Canada

**Keywords:** glomerulonephritis, Ontario, chronic kidney disease

## Abstract

**Purpose of Program:**

In 2018, Ontario Health (Ontario Renal Network) established a new multidisciplinary model of glomerulonephritis care to be available to all 27 Regional Renal Programs. This model of care was designed to fill existing gaps and ensure individuals with glomerulonephritis access to standardized, timely, and high-quality treatment close to home. This report describes the characteristics of individuals who received this care since it was established.

**Sources of Information:**

Provincial administrative health care databases.

**Methods:**

This is a descriptive study of the characteristics of adults with a registered glomerulonephritis visit provided by a multidisciplinary care team in Ontario, Canada between April 1, 2019, and March 31, 2023. Individuals were excluded if they had evidence of a kidney transplant prior to their first registered visit.

**Key Findings:**

A total of 6,926 individuals were included in the cohort. Every year since 2019, approximately 1,200 new individuals had a registered multidisciplinary visit. IgA nephropathy was the most common reported diagnosis at the first registered visit (1,407 of 6,926 [20.5%]). Over a median follow-up period of 2.7 years (interquartile range = 1.3-3.7) since their first registered visit, 420 individuals (6%) received kidney replacement therapy (maintenance dialysis or kidney transplant).

**Limitations:**

This description of individuals with registered visits underestimates the true prevalence of adults with glomerulonephritis in Ontario, as it does not capture those who did not register or who received more advanced disease management in other settings.

**Implications:**

The use of a new model of multidisciplinary glomerulonephritis care in Ontario, Canada is becoming well established. Ongoing analysis of administrative data will guide future healthcare planning and delivery.

## Résumé

**Objectif du programme:**

En 2018, Santé Ontario (Réseau rénal de l’Ontario) a établi un nouveau modèle multidisciplinaire de prise en charge de la glomérulonéphrite, destiné à être déployé dans les 27 programmes rénaux régionaux. Ce modèle a été élaboré afin de pallier les écarts dans les soins et de garantir à toute personne souffrant de glomérulonéphrite un accès uniforme, rapide et de qualité aux traitements, à proximité de chez elle. Ce rapport décrit les caractéristiques des personnes qui ont reçu ces soins depuis leur création.

**Sources de l’information:**

Bases de données provinciales administratives en santé.

**Méthodologie:**

Cette étude descriptive examine les caractéristiques des adultes de l’Ontario ayant eu une consultation documentée pour une glomérulonéphrite dispensée par une équipe de soins multidisciplinaire en Ontario entre le 1^er^ avril 2019 et le 31 mars 2023. Ont été exclues les personnes avec des preuves d’une greffe rénale avant leur première consultation documentée.

**Principaux résultats:**

Un total de 6 926 individus a été inclus dans la cohorte. Chaque année depuis 2019, environ 1 200 nouvelles personnes ont fait l’objet d’une visite multidisciplinaire documentée. La néphropathie à IgA représentait le diagnostic le plus courant lors de la première consultation documentée (1,407 sur 6,926 [20,5 %]). Après une période de suivi médiane de 2,7 ans (intervalle interquartile : 1,3 à 3,7) après leur première consultation documentée, 420 personnes (6 %) ont été prises en charge par thérapie de remplacement rénal (dialyse d’entretien ou greffe de rein).

**Limites:**

Cette analyse descriptive, fondée sur les consultations documentées, sous-estime la prévalence réelle de la glomérulonéphrite chez les adultes en Ontario, car elle ne tient pas compte des personnes non inscrites ou ayant bénéficié d’une prise en charge dans des milieux offrant des soins plus spécialisés.

**Conclusion:**

En Ontario, l’utilis ation d’un nouveau modèle de soins multidisciplinaires de la glomérulonéphrite est en train de s’implanter de manière durable. L’analyse continue des données administratives contribuera à orienter la planification et l’organisation futures des services de santé.

## Introduction

Glomerulonephritis (GN) is a heterogeneous group of kidney diseases that may cause progressive kidney damage. Despite being considered a rare disease, glomerular diseases account for approximately one in four cases of chronic kidney disease (CKD) worldwide, and one in five Canadians with prevalent end-stage kidney disease (ESKD).^1,2^ While GN can affect individuals of all ages, it often affects otherwise healthy young adults and is the primary diagnosis for approximately 40% of kidney transplant recipients aged 18 to 44 years in Canada.^2^

Despite its complexity, early identification and appropriate management can delay disease progression, reducing patient morbidity and health system costs. However, a 2015 provincial needs assessment found that GN care varied greatly across Ontario, Canada.^3^ Unstandardized GN practices across the province were seemingly underscored by factors such as a lack of provincial expertise in providing complex GN care, access to immunosuppressive medications, multidisciplinary team support and patient education.

Based on these findings, the first provincial strategy addressing gaps in GN care in Ontario was developed in 2018. The aim was to improve access to timely and effective multidisciplinary GN care.^3,4^ This report describes the characteristics of individuals who received care under this model since it was established.

## Methods

### About Ontario Health (Ontario Renal Network)

The Ontario Renal Network has been the Government of Ontario’s primary advisor on CKD since its establishment in 2009 (originally under Cancer Care Ontario). Now under Ontario Health, the Ontario Renal Network continues to manage the delivery of CKD services in the province by working in close partnership with 27 Regional Renal Programs, which are networks of hospitals and other agencies involved in providing kidney services at the local level, each falling within one of six larger Ontario Health Regions. Ontario Health (Ontario Renal Network) aims to support continuous improvement to Ontario’s kidney care system to provide person-centered and effective kidney care services in a safe, efficient, equitable and timely manner.^3-5^

### Ontario’s Multidisciplinary GN Program

Under the guidance of the GN Priority Panel (an expert panel including clinicians, administrators as well as patient and family advisors from across the province), Ontario Health (Ontario Renal Network) implemented a multidisciplinary GN model of care across all Regional Renal Programs in 2018 to address provincial gaps in GN care present at the time and to ensure people living with GN had access to standardized, timely and high-quality care close to home.^3^

While it is expected under the GN model of care that multidisciplinary care teams (nephrologists, nurses, dietitians, pharmacists, and social workers) are available in all Regional Renal Programs, six programs in particular host GN Specialty Clinics based on minimum volume and service requirements. The GN Specialty Clinics provide specialized renal care to complex patients and support local care in a shared-care model through partnerships with the surrounding 21 Regional Renal Programs to improve access to specialized GN care, streamline referrals across the province as needed and increase provincial expertise through professional education, training, and consultations. In other words, this care model was designed to support all Regional Renal Programs in Ontario through a “hub and spoke” system, linking them to a center of expertise to improve patient outcomes.

To receive funding, all Regional Renal Programs are expected to electronically report data on patients who receive multidisciplinary GN care in Ontario to the Ontario Renal Reporting System (ORRS). Electronic data reporting began in 2019. Reported data are linked to each multidisciplinary GN treatment event, where information on the diagnosis, corresponding diagnosis method, clinical characteristics (kidney function and proteinuria) and treatment information (hypertensive and immunosuppressive treatment, excluding doses) are collected.

### Data Sources

To summarize relevant population-based characteristics under Ontario’s multidisciplinary GN program, we conducted a descriptive analysis using administrative health care databases held at independent, nonprofit research institute(Ontario, Canada). ICES is an independent, nonprofit research institute whose legal status under Ontario’s health information privacy law allows it to collect and analyze health care and demographic data, without consent, for health system evaluation and improvement. The use of data in this project is authorized under section 45 of Ontario’s Personal Health Information Protection Act (PHIPA) and does not require review by a Research Ethics Board. Supplemental S1 and S2 contain information on databases and definitions discussed in this article. These datasets were linked using unique encoded identifiers and analyzed at ICES.

### Patient Population

This report describes adult Ontarians with a registered visit with a multidisciplinary GN care team in Ontario between April 1, 2019 (date of when electronic data collection began for the provincial multidisciplinary GN program in Ontario) and March 31, 2023. Patients were excluded if they were <18 years old, had missing or invalid demographic data, were a non-Ontario resident or had evidence of a kidney transplant prior to the date of first registered multidisciplinary GN visit during the observation window.

### Measures

Demographical (including age, sex, marginalization indices, fiscal index year and region) and select clinical characteristics (including primary diagnosis, diagnosis method, medications, and wait times for GN Specialty Clinic referrals) were evaluated as of the date of the first registered GN visit. Clinical comorbidities were evaluated over a five-year period prior to the visit date. Kidney function (measured by estimated glomerular filtration rate [eGFR]), proteinuria (measured by random urine albumin-to-creatinine ratio [ACR]) and general healthcare utilization (all-cause hospitalizations, emergency department visits, visits to a primary care provider and visits to a nephrologist) were evaluated in the one-year period prior to the first visit.

### Statistical Analyses

Continuous variables are presented as mean (standard deviation [SD]) or median (interquartile range [IQR]), as appropriate. Categorical variables are presented as frequency (percentage). Baseline characteristics of individuals who received kidney replacement therapy (maintenance dialysis or kidney transplant) during follow-up were compared with those of individuals who did not receive kidney replacement therapy. A standardized difference >10% was considered a meaningful difference.^6^ All analyses were conducted using SAS version 9.4 (SAS Institute Inc, Cary, North Carolina).

## Key Findings

### Demographic Characteristics

A total of 6,926 individuals were included in the cohort (Supplemental S3). Between April 1, 2019, and March 31, 2023, 24 of 27 renal programs reported multidisciplinary GN visits. Sixty percent of the entire cohort (n = 4,184) had a first registered visit at one of the six GN specialty clinics of which 2,305 were new referrals with an average wait time of 45 days between referral and first visit (IQR = 18-97). Every year since 2019, approximately 1200 new individuals were registered as receiving a multidisciplinary GN visit.

The median age of the entire cohort was 53 (IQR = 37-66) years, where 29% was between the ages of 18 and 39 years and 45% was below the age of 50 (Table 1). Nearly 50% of the cohort was female, with slightly more males requiring kidney replacement therapy during follow-up (54% compared with 46% of females).

**Table 1.** Baseline Demographic Characteristics.

Characteristic	Entire cohort (n = 6,926)	No KRT during follow-up (n = 6,506)	KRT during follow-up (n = 420)
Age, Years			
Median (IQR)	53 (37 - 66)	52 (37 - 66)	54 (39 - 66)
18-39	2,028 (29.3%)	1,920 (29.5%)	108 (25.7%)^a^
40-49	1,093 (15.8%)	1,024 (15.7%)	69 (16.4%)^a^
50-59	1,243 (17.9%)	1,166 (17.9%)	77 (18.3%)^a^
60-69	1,330 (19.2%)	1,242 (19.1%)	88 (21.0%)^a^
70-79	910 (13.1%)	848 (13.0%)	62 (14.8%)^a^
80+	322 (4.6%)	306 (4.7%)	16 (3.8%)^a^
Female, n (%)	3,450 (49.8%)	3,257 (50.1%)	193 (46.0%)^a^
Location, n (%)			
Rural	747 (10.8%)	701 (10.8%)	46 (11.0%)^a^
Missing	20 (0.3%)	NR	NR
Neighborhood Income Quintile, n (%)			
1 (least)	1,358 (19.6%)	1,261 (19.4%)	97 (23.1%)^a^
2	1,364 (19.7%)	1,271 (19.5%)	93 (22.1%)^a^
3 (mid)	1,400 (20.2%)	1,319 (20.3%)	81 (19.3%)^a^
4	1,401 (20.2%)	1,320 (20.3%)	81 (19.3%)^a^
5 (highest)	1,381 (19.9%)	1,315 (20.2%)	66 (15.7%)
Missing	22 (0.3%)	NR	NR
Marginalization: Households and Dwellings			
1 (least family and neighborhood instability)	1,550 (22.4%)	1,459 (22.4%)	91 (21.7%)^a^
2	1,240 (17.9%)	1,168 (18.0%)	72 (17.1%)^a^
3	1,237 (17.9%)	1,168 (18.0%)	69 (16.4%)^a^
4	1,294 (18.7%)	1,212 (18.6%)	82 (19.5%)^a^
5 (most family and neighborhood instability)	1,494 (21.6%)	1,397 (21.5%)	97 (23.1%)^a^
Missing	111 (1.6%)	102 (1.6%)	9 (2.1%)^a^
Marginalization: Material Resources			
1 (least poverty and inability to access and attain basic material needs)	1,359 (19.6%)	1,292 (19.9%)	67 (16.0%)
2	1,528 (22.1%)	1,450 (22.3%)	78 (18.6%)^a^
3	1,417 (20.5%)	1,332 (20.5%)	85 (20.2%)^a^
4	1,236 (17.8%)	1,144 (17.6%)	92 (21.9%)
5 (most poverty and inability to access and attain basic material needs)	1,275 (18.4%)	1,186 (18.2%)	89 (21.2%)^a^
Missing	111 (1.6%)	102 (1.6%)	9 (2.1%)^a^
Marginalization: Age and Labor Force			
1 (least amount of disability and dependence)	1,739 (25.1%)	1,625 (25.0%)	114 (27.1%)^a^
2	1,317 (19.0%)	1,256 (19.3%)	61 (14.5%)
3	1,244 (18.0%)	1,168 (18.0%)	76 (18.1%)^a^
4	1,221 (17.6%)	1,144 (17.6%)	77 (18.3%)^a^
5 (most amount of disability and dependence)	1,294 (18.7%)	1,211 (18.6%)	83 (19.8%)^a^
Missing	111 (1.6%)	102 (1.6%)	9 (2.1%)^a^
Marginalization: Racialized and Newcomer Populations			
1 (least racialized and newcomer populations)	1,089 (15.7%)	1013 (15.6%)	76 (18.1%)^a^
2	1,051 (15.2%)	987 (15.2%)	64 (15.2%)^a^
3	1,157 (16.7%)	1095 (16.8%)	62 (14.8%)^a^
4	1,384 (20.0%)	1309 (20.1%)	75 (17.9%)^a^
5 (most racialized and newcomer populations)	2,134 (30.8%)	2000 (30.7%)	134 (31.9%)^a^
Missing	111 (1.6%)	102 (1.6%)	9 (2.1%)
Fiscal Index Year, n (%)			
2019	3,334 (48.1%)	3,074 (47.2%)	260 (61.9%)
2020	1,219 (17.6%)	1,136 (17.5%)	83 (19.8%)^a^
2021	1,163 (16.8%)	1,115 (17.1%)	48 (11.4%)
2022	1,210 (17.5%)	1,181 (18.2%)	29 (6.9%)

*Note.* IQR: interquartile range; KRT: kidney replacement therapy.

a Standardized difference between no KRT and KRT groups >10%.

Approximately 11% of the cohort (n = 747) resided in rural Ontario. Individuals commonly resided in census areas with high and low levels of family and neighborhood instability (household and dwellings index; 22% in both the lowest and highest quintile), while 30.8% resided in census areas with the highest percentage of immigrants and/or people identifying as visible minorities (racialized and newcomer populations index).

### Clinical Characteristics

**Figure 1.**
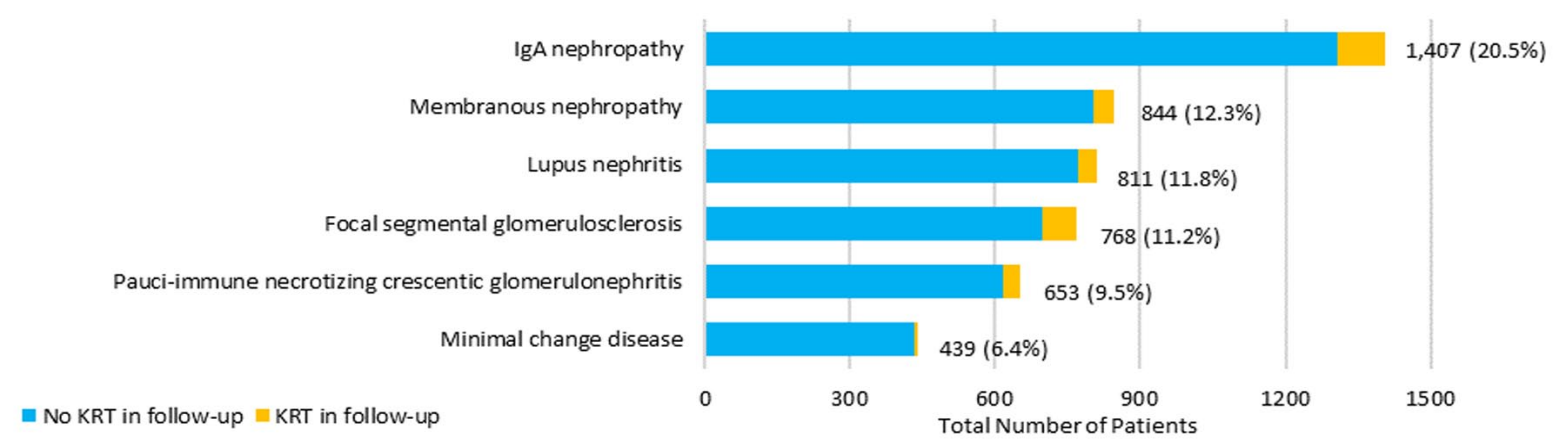
Most common etiologies of glomerulonephritis. *Note.* Where KRT is kidney replacement therapy.

At the time of their first registered multidisciplinary visit, nearly 80% of the cohort had been reportedly diagnosed by a kidney biopsy (n = 5,401) while 9% were diagnosed by serology. IgA Nephropathy was the most reported diagnosis at the time of the first registered visit (21%, n = 1,407) (Figure 1, Supplemental S4). Twelve percent of the cohort (n = 844) had a diagnosis of membranous nephropathy and 12% (n = 811) lupus nephritis.

Many had preserved kidney function (48% with eGFR>60) and high proteinuria (39% with urinary albumin-to-creatine ratio of >100 mg/mmol) at the time of their first registered GN visit (Table 2). Approximately 4% of individuals (n = 289) had a history of any dialysis treatment prior to their first registered GN visit.

**Table 2.** Baseline Clinical Characteristics and Healthcare Service Utilization.

Characteristic	Entire cohort (n = 6,926)	No KRT during follow-up (n = 6,506)	KRT during follow-up (n = 420)
Wait Times for GN Specialty Clinic Visit from Time of Referral, days (median, IQR)*	45 (18 - 97)	46 (18 - 98)	29 (13 - 73)
eGFR at Index, mL/min/1.73 m^2^			
Median (IQR)	57 (35 to 89)	60 (38 to 91)	23 (15 to 35)
>60	3,287 (47.5%)	3,256 (50.0%)	31 (7.4%)
45 to <60	1,063 (15.3%)	1,036 (15.9%)	27 (6.4%)
30 to <45	1,325 (19.1%)	1,247 (19.2%)	78 (18.6%)^a^
15 to <30%	993 (14.3%)	819 (12.6%)	174 (41.4%)
<15	252 (3.6%)	143 (2.2%)	109 (26.0%)
Missing	6 (0.1%)	NR	NR
Proteinuria at Index (UACR), mg/mmol			
Median (IQR)	60 (9 to 199)	55 (8 to 183)	202 (72 to 401)
<3	1,059 (15.3%)	1,045 (16.1%)	14 (3.3%)
3 to <30	1,564 (22.6%)	1,530 (23.5%)	34 (8.1%)
30 to <100	1,432 (20.7%)	1,347 (20.7%)	85 (20.2%)^a^
100+	2,693 (38.9%)	2,417 (37.2%)	276 (65.7%)
Missing	178 (2.6%)	167 (2.6%)	11 (2.6%)
History of Dialysis Treatment	289 (4.2%)	246 (3.8%)	43 (10.2%)
Comorbidities			
Chronic obstructive pulmonary disease	864 (12.5%)	789 (12.1%)	75 (17.9%)
Congestive heart failure	563 (8.1%)	505 (7.8%)	58 (13.8%)
Coronary artery disease (including angina)	1,080 (15.6%)	993 (15.3%)	87 (20.7%)
Deep Vein Thrombosis	202 (2.9%)	189 (2.9%)	13 (3.1%)
Dementia	77 (1.1%)	NR	NR
Diabetes	1,433 (20.7%)	1,319 (20.3%)	114 (27.1%)
Hypertension	3,903 (56.4%)	3,597 (55.3%)	306 (72.9%)
Major cancer	853 (12.3%)	795 (12.2%)	58 (13.8%)^a^
Myocardial Infarction	140 (2.0%)	125 (1.9%)	15 (3.6%)
Peripheral vascular disease	42 (0.6%)	NR	NR
Stroke/TIA	291 (4.2%)	275 (4.2%)	16 (3.8%)^a^
Charlson Comorbidity Index, over last 2 years			
Median (IQR)	0 (0 to 2)	0 (0 to 2)	2 (0 to 2)
0	5,311 (76.7%)	5,063 (77.8%)	248 (59.0%)
1	486 (7.0%)	459 (7.1%)	27 (6.4%)^a^
2	628 (9.1%)	546 (8.4%)	82 (19.5%)
3+	501 (7.2%)	438 (6.7%)	63 (15.0%)
Hospitalizations in the last year			
Median (IQR)	1 (1 to 2)	1 (1 to 2)	1 (1 to 2)
0 hospitalizations	5,002 (72.2%)	4,756 (73.1%)	246 (58.6%)
1 hospitalization	1,341 (19.4%)	1,230 (18.9%)	111 (26.4%)
2 hospitalizations	401 (5.8%)	367 (5.6%)	34 (8.1%)
3+ hospitalizations	182 (2.6%)	153 (2.4%)	29 (6.9%)
Emergency Department Visits in the last year			
Median (IQR)	2 (1 to 3)	2 (1 to 3)	2 (1 to 3)
0 visits	3,660 (52.8%)	3,492 (53.7%)	168 (40%)
1 visit	1,553 (22.4%)	1,449 (22.3%)	104 (24.8%)^a^
2 visits	755 (10.9%)	694 (10.7%)	61 (14.5%)
3+ visits	958 (13.8%)	871 (13.4%)	87 (20.7%)
Visits to a Primary Care Provider in the last year			
Median (IQR)	6 (3 to 11)	6 (3 to 11)	6 (3 to 12)
0 visits	545 (8.6%)	515 (8.6%)	30 (7.9%)^a^
1-2 visits	1,244 (19.6%)	1,170 (19.6%)	74 (19.4%)^a^
3-5 visits	1,054 (16.6%)	993 (16.6%)	61 (16.0%)^a^
6+ visits	3,511 (55.3%)	3,294 (55.2%)	217 (56.8%)^a^
Visits to a Nephrologist in the last year			
Median (IQR)	3 (1 to 4)	3 (1 to 4)	4 (3 to 7)
0 visits	1,247 (19.7%)	1,215 (20.4%)	32 (8.9%)
1-2 visits	2,725 (43.1%)	2,635 (44.2%)	90 (25.1%)
3-5 visits	1,352 (21.4%)	1,251 (21.0%)	101 (28.2%)
6+ visits	1,002 (15.8%)	867 (14.5%)	135 (37.7%)

*Note.* eGFR: Estimated glomerular filtration rate; GN: Glomerulonephritis; IQR: Interquartile range; KRT: Kidney replacement therapy; TIA: Transient ischemic attack; UACR: Urinary Albumin-to-Creatine Ratio.

* Includes only individuals with a reported new referral visit to a GN Specialty Clinic.

a Standardized difference between no KRT and KRT groups >10%.

**Figure 2.**
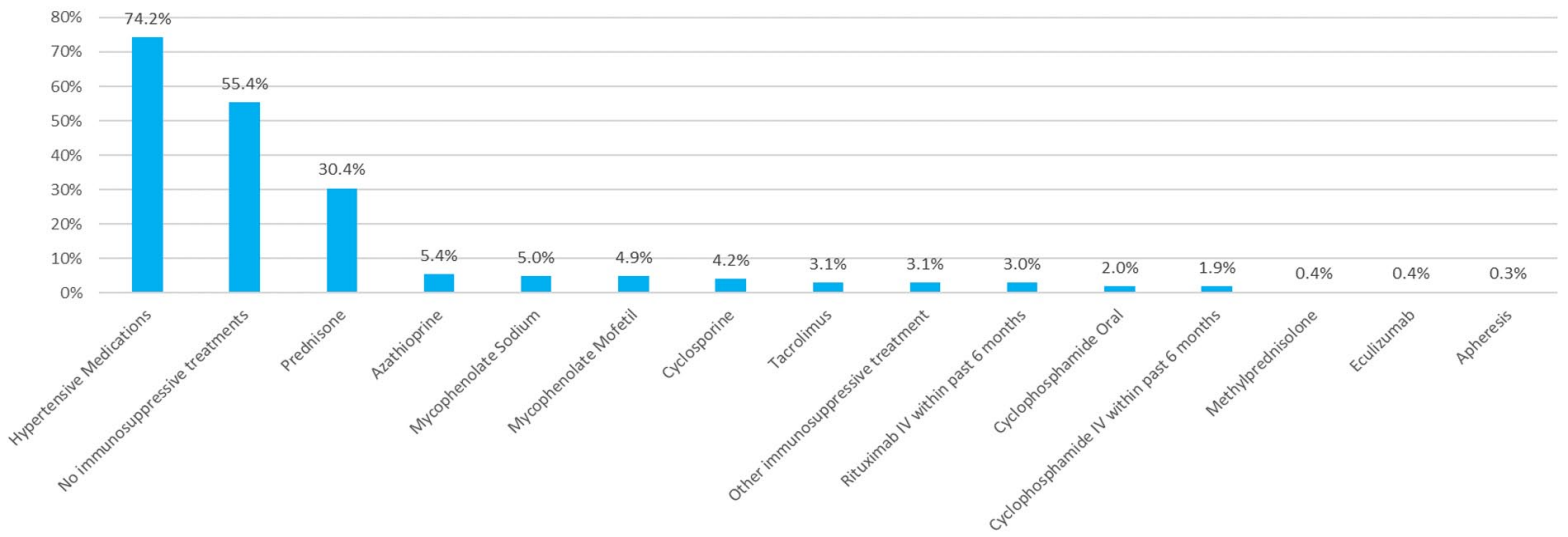
Treatment profile at baseline. *Note.* Individuals could be counted for multiple times between types of immunosuppressive treatments.

Hypertension, diabetes, coronary artery disease, chronic obstructive pulmonary disease, and cancer were common comorbidities at the time of first registered visit. Nearly 75% of individuals were receiving hypertensive medications, primarily blockers of the renin angiotensin system at the time of their first registered multidisciplinary GN visit (n = 5,123) (Figure 2). Forty-five percent (n = 3,098) of the cohort were being treated with immunosuppressive therapy at the time of the first registered GN visit, with prednisone being the most common reported treatment (n = 2,097, 30%).

### Healthcare Service Utilization

Nearly 30% (n = 1,924) of the cohort had at least one hospitalization for any reason in the year prior to their first registered multidisciplinary GN visit (Table 2). Nearly 50% had at least one emergency department visit (n = 3,266). Less than 10% of the cohort had not seen a primary care provider in the year prior to their first registered multidisciplinary GN visit (n = 545), while 55% of individuals had greater than five visits (n = 3,511). Nearly 20% had not seen a nephrologist in the year prior to the first registered visit (n = 1,247).

Over a median follow-up period of 2.7 (IQR = 1.3-3.7) years since their first registered multidisciplinary GN visit, 420 individuals (6%) received kidney replacement therapy (dialysis or transplant; 2.6 events per 100 patient years), 354 individuals (5%) died (2.1 events per 100 patient years) and 127 individuals (2%) emigrated from the province (0.8 events per 100 patient years). Hypertension, diabetes, coronary artery disease, and chronic obstructive pulmonary disease were more prevalent in individuals who required kidney replacement therapy compared with those who did not. Individuals who received kidney replacement therapy had lower kidney function and greater proteinuria at their first visit (67% with eGFR <30; 66% with urinary albumin-to-creatinine ratio of >100 mg/mmol), 10% of which (n = 43) had a history of dialysis treatment.

## Discussion

**Figure 3.**
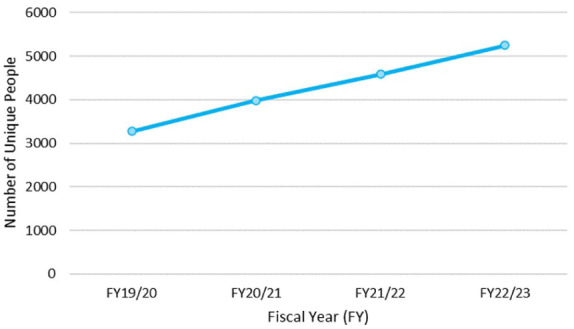
Prevalence of individuals with a multidisciplinary visit under the GN model of care. *Note.* Between April 1, 2019 and March 31, 2023; courtesy of Ontario Health (Ontario Renal Network). Ontario Health (Ontario Renal Network), unpublished internal report, 2023. Permission granted 2025.

In 2018, Ontario Health (Ontario Renal Network) established a new multidisciplinary model of GN care to be available to all 27 Regional Renal Programs. It was successfully launched and use of this model of care in Ontario is becoming well established, with nearly 7,000 adult Ontarians registered within the first 4 years of implementation (Figure 3).

Relative to other reported cohorts, adults with registered multidisciplinary GN visits in Ontario are similar in age to adults diagnosed with primary GN in British Columbia, Canada, Spain and Denmark at the time of their biopsy, as well as individuals with medical claims for GN under the Optum Clinformatics Employer Group Health Plan in the United States of America.^7-10^ Compared with other population-based estimates, the ratio of females to males in the Ontario cohort may be more balanced.^9,10^ In Canada specifically, a key difference between the Ontario and British Columbia provincial GN datasets is that British Columbia centralizes all provincial biopsy information with detailed reports on diagnoses and pathology characteristics, whereas Ontario collects diagnosis and treatment data at the time of a reported multidisciplinary GN visit.^11^ Therefore, detailed renal histopathology cannot be determined from the Ontario GN dataset. However, consistent with global evidence, and evidence from British Columbia, IgA nephropathy is the most reported GN diagnosis, as also noted in Ontario.^12,13^

Although overall the entire cohort seems balanced in terms of income quintiles, more individuals who required kidney replacement therapy during follow-up had lower neighborhood income quintiles and were from areas that were generally more marginalized, consistent with previous findings.^14^ Population-based data from British Columbia, Canada, suggest select etiologies of GN may cluster in sparsely populated regions with limited access to care, where incidence may be inversely associated with socioeconomic status.^15,16^ However in Ontario, previous reported lack of expertise for complex GN cases, and variable timing to access immunosuppressive medications^3,17^ likely also factored toward worse patient outcomes.

## Limitations

Our study has a few limitations. First, our estimate of individuals with registered GN visits underestimates the true prevalence of individuals with GN in Ontario. As the provincial GN program captures eligible multidisciplinary nephrology care visits only, those not receiving multidisciplinary GN care in Ontario are not funded under this program, and therefore, not captured, and we know that not all physicians have transitioned their patients to multidisciplinary clinics. Second, this dataset does not capture people with GN with more advanced diseases enrolled and receiving care in Multi-Care Kidney Clinics in Ontario, who have GN as the etiology of their advanced CKD, but did not have GN-specific visits registered.

## Implications

The multidisciplinary GN model of care is continuing to grow in Ontario, Canada. As more community renal programs expand implementation of the model of care, and the cohort size and vintage of this dataset continues to mature, further longitudinal analyses may inform (1) trends in incidence and prevalence rates of various GN pathologies in Ontario; (2) whether varying regional and sociodemographic characteristics are associated with access to multidisciplinary GN care and better outcomes; and (3) any changes to treatment prescribing patterns which may be reflective of growing GN expertise and changes to medication access. Such ongoing analysis of the administrative data will guide future healthcare planning and delivery.

## Supplemental Material

sj-docx-1-cjk-10.1177_20543581251394082 – Supplemental material for A New Multidisciplinary Model of Glomerulonephritis Care in Ontario: A Descriptive Program Report Supplemental material, sj-docx-1-cjk-10.1177_20543581251394082 for A New Multidisciplinary Model of Glomerulonephritis Care in Ontario: A Descriptive Program Report by Arrti A. Bhasin, Stephanie N. Dixon, Lavanya Bathini, Nivethika Jeyakumar, Lima F. Rodrigues, Yuguang Kang, Peter G. Blake, Amit X. Garg and Michelle A. Hladunewich in Canadian Journal of Kidney Health and Disease
