# Supplementary material for: A New Multidisciplinary Model of Glomerulonephritis Care in Ontario: A Descriptive Program Report
Source: Can J Kidney Health Dis. 2025 Dec 1;12:20543581251394082. doi: 10.1177/20543581251394082 (PMC12669530; doi:10.1177/20543581251394082)
Supplement: sj-docx-1-cjk-10.1177_20543581251394082 – Supplemental material for A New Multidisciplinary Model of Glomerulonephritis Care in Ontario: A Descriptive Program Report [file sj-docx-1-cjk-10.1177_20543581251394082.docx]

**Supplemental S1**

**Administrative Data Sources**

| **Database** | **Description** | **Use in Study** |
| --- | --- | --- |
| Canadian Institute for Health Information’s Discharge Abstract Database/ Same Day Surgery (CIHI-DAD/ SDS) | Contains administrative, clinical (diagnosis and procedures/ interventions) and demographic information for all admission to acute care hospitals. | Hospitalizations including procedures and diagnoses for descriptive characteristics. |
| Chronic Obstructive Pulmonary Disease (COPD) | Information on all individuals in Ontario with chronic obstructive pulmonary disease using a validated algorithm of physician billing claims and hospital or same day surgery records. | Patients with a history of chronic obstructive pulmonary disease for the descriptive characteristics. |
| ICES Physician Database (IPDB) | Information on all physicians who have practiced in Ontario and contains information on demographics, specialty, location of practice and measures of physician activity. | Previous physician and nephrology visits for the descriptive characteristics. |
| Canadian Institute for Health Information’s National Ambulatory Care Reporting System (CIHI-NACRS) | Contains administrative, clinical (diagnosis and procedures) and demographic information for all patient visits made to hospital- and community-based ambulatory care centres (emergency departments, day surgery units, hemodialysis units, and cancer care clinics). | Previous emergency department visits including diagnoses for the descriptive characteristics. |
| Ontario Dementia Database (DEMENTIA) | Information on all individuals in Ontario who have been identified with Alzheimer’s and related dementias using a validated algorithm of physician billing claims, hospital or same day surgery records, or drug claims. | Patients with a history of dementia for the descriptive characteristics. |
| Ontario Diabetes Dataset (ODD) | Information on all individuals in Ontario with any type of non-gestational diabetes using a validated algorithm of physician billing claims and hospital and same day surgery records. | Patients with a history of diabetes for the descriptive characteristics. |
| Ontario Health Insurance Plan (OHIP) | Contains information on inpatient and outpatient services provided to Ontario residents eligible for the province’s publicly funded health insurance system for fee-for-service health care practitioners and shadow billing for those paid through non-fee-for-service payment plans. | For the cohort creation, to exclude those with evidence of a kidney transplant and for previous physician and nephrology visits, procedures and diagnoses for the descriptive characteristics. |
| Ontario Hypertension Dataset (HYPER) | Information on all individuals in Ontario with hypertension using a validated algorithm of physician billing claims and inpatient hospital or same day surgery records. | Patients with a history of hypertension for the descriptive characteristics. |
| Ontario Laboratories Information System (OLIS) | Laboratory test orders and results from hospitals, community and public health laboratories. | Previous kidney function based on serum creatinine and urine albumin-to-creatinine ratio laboratory values for the descriptive characteristics. |
| Ontario Marginalization Index (ONMARG) | Geographic-based index developed to quantify the degree of marginalization occurring across Ontario, Canada. It is comprised of four dimensions thought to underlie the construct of marginalization. | Descriptive characteristics on households and dwellings, material resources, age and labour force and racialized and newcomer populations. |
| Ontario Renal Reporting System (ORRS) | Contains demographics, clinical and administrative information on individuals with chronic kidney disease and end-stage kidney disease. | For the cohort creation of people receiving multidisciplinary GN care in Ontario and to exclude those with evidence of a kidney transplant. Used to identify GN clinic characteristics including location of clinic, wait time from referral to clinic visit, GN diagnosis, diagnosis method and hypertensive/ immunosuppressive medication use. Also used to identify previous kidney function based on serum creatinine and urine albumin-to-creatinine ratio laboratory values and history of dialysis for the descriptive characteristics and dialysis during follow-up. |
| Registered Persons Database (RPDB) | Provides basic demographic information for those issued an Ontario health insurance number. The database also provides time periods for which an individual was eligible to receive publicly funded health insurance benefits and the best-known postal code for each resident. | For the cohort creation, data cleaning exclusions including date of death and non-Ontario residents. Additional information on date of birth, sex, rural/ urban location and income quintile for descriptive characteristics and emigration during follow-up. |

**Supplemental S2**

**Diagnostic and Procedural Codes**

| **Characteristics** | **Databases** | **Codes** |
| --- | --- | --- |
| Multidisciplinary GN care (Inclusion) | Ontario Renal Reporting System (ORRS) | TREATMENTCHANGECD: VG |
| Kidney transplant (Exclusion) | ORRS  Ontario Health Insurance Plan (OHIP) | TREATMENTCHANGECD: TX  OHIP fee code: S435, S434 |
| Dialysis  (Outcome) | ORRS  Canadian Institute for Health Information’s Discharge Abstract Database/ National Ambulatory Care Reporting System (CIHI-DAD/ NACRS)  OHIP | TREATMENTCHANGECD: N, M, TI  Canadian Classification of Health Interventions (CCI): 1PZ21, 1PZ21HPD4, 1PZ21HQBR, 1PZ21HQBS  OHIP fee code: 150, 153, 154, 155, 157, 162, 174, 175, 183, 185, 203, 204, 205, 206, 207, 208  OHIP diagnosis code: G082, G083, G085, G090, G091, G092, G093, G094, G095, G096, G294, G295, G323, G325, G326, G330, G331, G333, G860, G861, G862, G863, G864, G865, G866, H540, H740, R849 |

**Supplemental S3**

**Cohort Flow Diagram**

**Supplemental S4**

**Baseline diagnosis and treatment characteristics**

| Characteristic | Entire Cohort  (n=6,926) | No KRT During Follow-Up  (n=6,506) | KRT During Follow-Up  (n=420) |
| --- | --- | --- | --- |
| **Primary Diagnosis** | | | |
| Alport nephritis | 53 (0.8%) | NR | NR |
| Amyloidosis | 76 (1.1%) | 67 (1.0%) | 9 (2.1%)* |
| Anti-glomerular basement membrane glomerulonephritis | 28 (0.4%) | 22 (0.3%) | 6 (1.4%) |
| C3 glomerulopathy | 62 (0.9%) | 53 (0.8%) | 9 (2.1%) |
| Cryoglobulinemic glomerulonephritis | 8 (0.1%) | 8 (0.1%) | 0 (0.0%)* |
| Diabetic nephropathy | NR | NR | NR |
| Fabry disease | 9 (0.1%) | 9 (0.1%) | 0 (0.0%)* |
| Fibrillary glomerulonephritis | 41 (0.6%) | 35 (0.5%) | 6 (1.4%)* |
| Focal segmental glomerulosclerosis | 768 (11.2%) | 697 (10.8%) | 71 (16.9%) |
| Global and segmental glomerulosclerosis | 75 (1.1%) | NR | NR |
| IgA nephropathy | 1,407 (20.5%) | 1,309 (20.3%) | 98 (23.4%)* |
| Immunotactoid glomerulopathy | 12 (0.2%) | NR | NR |
| Acute interstitial nephritis | 49 (0.7%) | 49 (0.8%) | 0 (0.0%) |
| Lupus nephritis | 811 (11.8%) | 772 (12.0%) | 39 (9.3%)* |
| Immune complex mediated membranoproliferative glomerulonephritis | 142 (2.1%) | 127 (2.0%) | 15 (3.6%) |
| Membranous nephropathy | 844 (12.3%) | 804 (12.5%) | 40 (9.5%) |
| Minimal change disease | 439 (6.4%) | 432 (6.7%) | 7 (1.7%) |
| Monoclonal immunoglobulin deposition disease | 22 (0.3%) | NR | NR |
| Pauci-immune necrotizing crescentic glomerulonephritis | 653 (9.5%) | 615 (9.5%) | 38 (9.1%)* |
| Post-infectious glomerulonephritis | 23 (0.3%) | 23 (0.4%) | 0 (0.0%)* |
| IgA dominant post-infectious  glomerulonephritis | 16 (0.2%) | NR | NR |
| Proliferative glomerulonephritis with monoclonal immunoglobulin deposits | 8 (0.1%) | 8 (0.1%) | 0 (0.0%)* |
| Proliferative glomerulonephritis with prominent C3 deposition | NR | NR | NR |
| Immune complex mediated proliferative glomerulonephritis | 43 (0.6%) | 37 (0.6%) | 6 (1.4%)* |
| Structural abnormality of the glomerular basement membrane | 20 (0.3%) | NR | NR |
| Thin basement membrane nephropathy | 36 (0.5%) | NR | NR |
| Thrombotic microangiopathy | 63 (0.9%) | 56 (0.9%) | 7 (1.7%)* |
| Crescentic glomerulonephritis | 29 (0.4%) | NR | NR |
| Inadequate for diagnosis | NR | NR | NR |
| Not yet diagnosed | 272 (4.0%) | NR | NR |
| Other | 851 (12.4%) | 801 (12.4%) | 50 (11.9%)* |
| **Treatment** | | | |
| Hypertensive Treatment | 5,123 (74.2%) | 4,761 (73.4%) | 362 (86.4%) |
| Apheresis | 19 (0.3%) | 19 (0.3%) | 0 (0.0%)* |
| Azathioprine | 374 (5.4%) | 356 (5.5%) | 18 (4.3%)* |
| Cyclophosphamide IV within past 6 months | 134 (1.9%) | 122 (1.9%) | 12 (2.9%)* |
| Cyclophosphamide Oral | 135 (2.0%) | 122 (1.9%) | 13 (3.1%)* |
| Cyclosporine | 293 (4.2%) | 264 (4.1%) | 29 (6.9%)* |
| Methylprednisolone | 26 (0.4%) | NR | NR |
| Mycophenolate Mofetil | 335 (4.9%) | 313 (4.8%) | 22 (5.3%)* |
| Mycophenolate Sodium | 345 (5.0%) | 316 (4.9%) | 29 (6.9%)* |
| Prednisone | 2,097 (30.4%) | 1,939 (29.9%) | 158 (37.7%) |
| Rituximab IV within past 6 months | 208 (3.0%) | 193 (3.0%) | 15 (3.6%)* |
| Tacrolimus | 214 (3.1%) | 204 (3.1%) | 10 (2.4%)* |
| Eculizumab | 26 (0.4%) | NR | NR |
| No immunosuppressive treatments | 3,828 (55.4%) | 3,635 (56.0%) | 193 (46.1%) |
| Other | 215 (3.1%) | 206 (3.2%) | 9 (2.1%)* |

IgA: Immunoglobulin A; IV: Intravenous; KRT: Kidney replacement therapy; NR: Not Reported due to small cells (n<6)

*Standardized difference between no KRT and KRT groups >10%
